# Isolation of Intact Mitochondria From *Drosophila melanogaster* and Assessment of Mitochondrial Respiratory Capacity Using Seahorse Analyzer

**DOI:** 10.21769/BioProtoc.5180

**Published:** 2025-02-05

**Authors:** Christopher M. Groen, Anthony J. Windebank

**Affiliations:** Department of Neurology, Mayo Clinic, Rochester, NY, USA;

**Keywords:** Mitochondria, Respiration, Drosophila, Seahorse, Differential centrifugation, Oxygen consumption

## Abstract

Analysis of mitochondrial function has broad applicability in many research specialties. Neurodegenerative disorders such as chemotherapy-induced peripheral neuropathy (CIPN) often exhibit damaged mitochondria or reduced mitochondrial respiratory capacity. Isolation of intact mitochondria for protein analysis or respiration measurements has been previously reported in numerous model organisms. Here, we describe an adaptation of previous protocols to isolate intact functional mitochondria from *Drosophila melanogaster* for use in a model of CIPN. Whole *Drosophila* are ground in isolation buffer, and mitochondria are purified using differential centrifugation through a sucrose and mannitol solution. The intact mitochondria are plated as a monolayer for measurements of mitochondrial oxygen consumption rates and response to inhibitor compounds on an Agilent Seahorse analyzer. This experimental protocol is quick and yields a purified population of intact mitochondria that may be used for functional assays for several hours after isolation. The isolated mitochondria may be used for respiration measurements, which reflect their health, and stored for protein or genetic analysis. Mitochondrial populations from multiple strains or treatment groups can be easily compared simultaneously. The rapid biochemical assessment of mitochondria, in combination with the utility of *Drosophila* as an in vivo genetic model system, offers great potential for researchers to probe the impact of genetics and pharmacologic interventions on mitochondrial respiratory capacity.

Key features

• This protocol describes rapid isolation of intact, functional mitochondria that may be used for respiration measurements or other biochemical analyses.

• Mitochondria isolated from *Drosophila* are assessed in an Agilent Seahorse analyzer utilizing multiple substrates and electron transport chain inhibitors to fully characterize mitochondrial respiratory capacity.

• This protocol is optimized to use *Drosophila* for easy in vivo genetic and pharmacologic manipulation, and assessment of the impact on mitochondrial function.

## Graphical overview


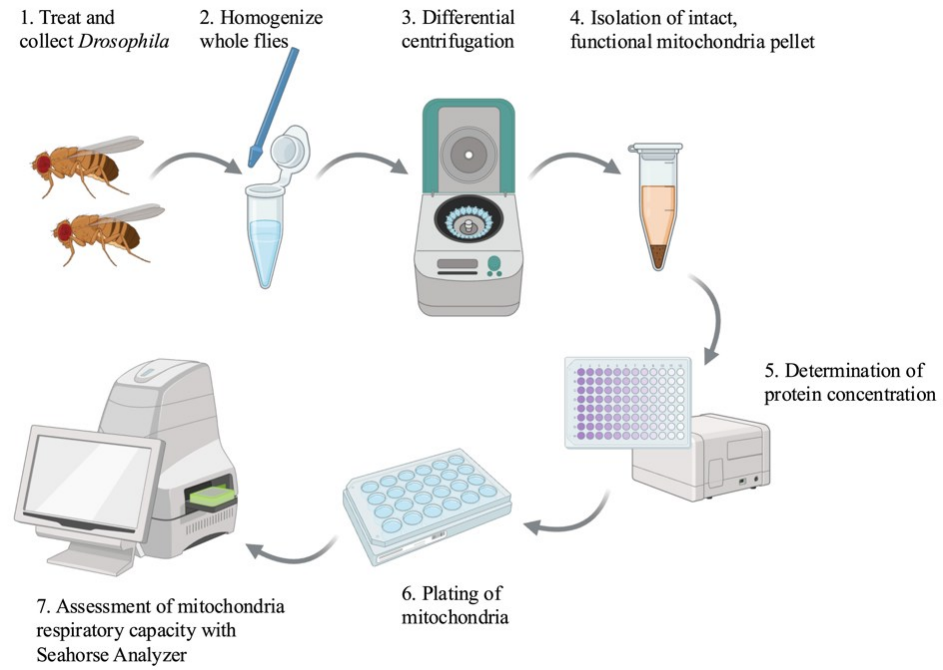


**Created in** BioRender.com

## Background

Mitochondria function is increasingly recognized as an essential factor in human health and disease. Altered mitochondria function is observed in cancer [1], aging [2–4], metabolic disorders [5], and neurodegenerative disorders such as Parkinson’s Disease [6] and chemotherapy-induced neuropathy [7,8]. Assessment of mitochondrial function and cellular respiration rates can reveal disease states in an organism or tissue. These measurements are invaluable tools to parse the reactions of cells to acute toxic stressors, genetic manipulations, or potential preventive and therapeutic interventions [9]. Analysis of mitochondrial health has progressed from earlier techniques using Clark electrodes to measure O_2_ consumption in individual samples to more advanced instrumentation such as Agilent Seahorse analyzers, which are capable of simultaneous analysis of 96-well plates. Modern respiration analyzers can also measure real-time response to the addition of substrates, metabolites, and mitochondria inhibitors. Mitochondria respiration analysis has been measured using intact cells in cell culture plates, dissociated tissues, and isolated mitochondria. Insights gained from these experiments advance our understanding of the role of mitochondria in disease and provide new potential biomarkers for the assessment of disease states [9].

Previous publications have described detailed protocols for the measurement of cellular respiration [9–11], isolation of mitochondria for metabolic analysis [12], and use of isolated mitochondria from mouse muscle tissue for oxygen consumption rate analysis in Seahorse analyzers [13]. *Drosophila*-specific protocols in this field include detailed experimental methods for isolation of larval mitochondria [14], metabolic analysis of flight muscle mitochondria [12], and isolation of mitochondria for enzymatic analysis of electron transport chain components [15]. The protocol described here is a modification of previous mitochondria isolation procedures from *Drosophila*, with optimization of Seahorse analysis of these isolated mitochondria to assess differences in respiratory capacity between different fly strains treated with chemotherapy agents known to damage mitochondria. This protocol was previously described in a manuscript reporting differences in cisplatin sensitivity between *Drosophila* strains and showed that the mitochondria have different basal respiration rates and different abilities to maintain activity following treatment with toxic stressors like cisplatin [16].

The protocol described here allows for rapid isolation of a pure mitochondria pellet. The mitochondria retain their activity for several hours after isolation if kept on ice. In addition, the intact mitochondria may be used for protein analysis or enzymatic activity assays. Isolation of mitochondria allows for direct assessment of electron transport chain function and the impact of multiple substrates and inhibitors. This allows researchers to focus on specific aspects of cell metabolism without the variables presented by whole-cell metabolomics. The availability of substrates can be tightly controlled by the user, whereas whole-cell or tissue respiration measurements must consider the availability of metabolites contributed by the whole-cell environment. ADP is often used as an injection compound to measure mitochondrial respiration before and after the substrate for ATP synthase is available. Respiration through Complex I of the electron transport chain may be measured through the use of pyruvate and malate as initial substrates. Succinate can be used as a substrate for Complex II, and ascorbate is a substrate for Complex IV. Likewise, inhibitors block components of the electron transport chain. Rotenone and antimycin A block Complex I and Complex III, respectively. Oligomycin blocks the activity of ATP synthase, while FCCP uncouples oxygen consumption from ATP synthesis. Careful selection of substrates and inhibitors can reveal a great deal about the function of isolated mitochondria. This method does not, however, provide a complete picture of cellular respiration, as the mitochondria are removed from the context of the cell. The lack of whole cells limits data collection from the Seahorse to oxygen consumption rate (OCR). The extracellular acidification rate (ECAR) measured by the Seahorse as a readout of glycolytic activity is not useful when only assessing isolated mitochondria. Researchers should carefully choose an experimental protocol based on specific goals in characterizing cellular metabolism.

## Materials and reagents

**Biological materials**

1. *Drosophila melanogaster* stocks (Bloomington Drosophila Stock Center, stock numbers 36303 and 36304 were used in the sample data)

**Reagents**

1. Sucrose (Sigma-Aldrich, catalog number: S1888-1KG)

2. D-Mannitol (Sigma-Aldrich, catalog number: M4125-100G)

3. HEPES buffer, 1 M, pH 7.2 (Corning, catalog number: 25-060-Cl)

4. EDTA, 0.5 M (Sigma-Aldrich, catalog number: 03690-100ML)

5. Bovine serum albumin (BSA) (Sigma-Aldrich, catalog number: 05470-5G)

6. Potassium hydroxide (KOH) (Sigma-Aldrich, catalog number: 221473-25G)

7. Monobasic potassium phosphate (KH_2_PO_4_) (Sigma-Aldrich, catalog number: 1551139-5G)

8. Magnesium chloride (MgCl_2_) (Sigma-Aldrich, catalog number: M8266-100G)

9. EGTA, 0.5 M (RPI, catalog number: E14100-50.0)

10. Pyruvic acid (Sigma-Aldrich, catalog number: 107360-25G)

11. Malic acid (Sigma-Aldrich, catalog number: M6413-25G)

12. Adenosine 5’-diphosphate monopotassium salt dihydrate (ADP) (Sigma-Aldrich, catalog number: A5285)

13. Oligomycin A (Sigma-Aldrich, catalog number: 75351)

14. Carbonyl cyanide 4-(trifluoromethoxy) phenylhydrazone (FCCP) (Sigma-Aldrich, catalog number: C2920)

15. Rotenone (Sigma-Aldrich, catalog number: R8875)

16. Antimycin A (Sigma-Aldrich, catalog number: A8674)

17. Seahorse XF calibrant (Agilent, catalog number: 100840-000)

18. DC Protein Assay Kit I (Bio-Rad, catalog number: 5000111)

19. 95% Ethanol (Millipore-Sigma, catalog number: 65348-M)

**Solutions**

1. 10% w/v BSA (see Recipes)

2. Pyruvate stock solution (see Recipes)

3. Malate stock solution (see Recipes)

4. ADP stock solution (see Recipes)

5. Oligomycin A stock solution (see Recipes)

6. FCCP stock solution (see Recipes)

7. Rotenone stock solution (see Recipes)

8. Antimycin A stock solution (see Recipes)

9. Mitochondria isolation buffer (see Recipes)

10. Mitochondria assay buffer (2× stock) (see Recipes)

11. Working mitochondria assay buffer (1×) with substrates (see Recipes)

**Recipes**

**1. 10% w/v BSA**

**Table:** 

Reagent	Final concentration	Quantity or Volume
BSA	10% w/v	1 g
ddH_2_O	n/a	*
Total (optional)	n/a	10 mL

*Fully dissolve BSA in ~5–7 mL of ddH_2_O and then bring the solution to a final volume of 10 mL.

Aliquot and store at -20 °C.

**2. Pyruvate stock solution (100 mM)**

**Table:** 

Reagent	Final concentration	Quantity or Volume
Pyruvic acid	100 mM	0.8806 g
ddH_2_O	n/a	100 mL
Total (optional)	n/a	100 mL

Aliquot and store at -20 °C.

**3. Malate stock solution (100 mM)**

**Table:** 

Reagent	Final concentration	Quantity or Volume
Malic acid	100 mM	1.34 g
95% ethanol	n/a	100 mL
Total (optional)	n/a	100 mL

Aliquot and store at -20 °C.

**4. ADP stock solution (100 mM)**

**Table:** 

Reagent	Final concentration	Quantity or Volume
ADP	100 mM	5.0132 g
ddH_2_O	n/a	100 mL
Total (optional)	n/a	100 mL

Aliquot and store at -20 °C.

**5. Oligomycin A stock solution (10 mM)**

**Table:** 

Reagent	Final concentration	Quantity or Volume
Oligomycin A	10 mM	0.079 g
95% ethanol	n/a	10 mL
Total (optional)	n/a	10 mL

Aliquot and store at -20 °C.

**6. FCCP stock solution (10 mM)**

**Table:** 

Reagent	Final concentration	Quantity or Volume
FCCP	10 mM	0.025 g
95% ethanol	n/a	10 mL
Total (optional)	n/a	10 mL

Aliquot and store at -20 °C.

**7. Rotenone stock solution (5 mM)**

**Table:** 

Reagent	Final concentration	Quantity or Volume
Rotenone	5 mM	0.019 g
95% ethanol	n/a	10 mL
Total (optional)	n/a	10 mL

Aliquot and store at -20 °C.

**8. Antimycin A stock solution (10 mM)**

**Table:** 

Reagent	Final concentration	Quantity or Volume
Antimycin A	10 mM	0.055 g
95% ethanol	n/a	10 mL
Total (optional)	n/a	10 mL

Aliquot and store at -20 °C.

**9. Mitochondria isolation buffer**

**Table:** 

Reagent	Final concentration	Quantity or Volume
Sucrose	70 mM	4.79 g
D-Mannitol	210 mM	7.65 g
1 M HEPES, pH 7.2	5 mM	1 mL
0.5 M EDTA	1 mM	0.4 mL
10% BSA	0.5%	10 mL*
KOH	n/a	pH to 7.2
ddH2O	n/a	**
Total (optional)	n/a	200 mL

*BSA is included in the isolation buffer and assay buffers to bind free fatty acids released during tissue homogenization and helps to maintain mitochondrial membrane potential and respiratory function.

**Fully dissolve solid reagents in ~100 mL of ddH_2_O. Use KOH to bring the pH to 7.2 and then bring the solution to a final volume of 200 mL.

Aliquot and store at -20 °C

**10. Mitochondria assay buffer (2× stock)**

**Table:** 

Reagent	Final concentration	Quantity or Volume
Sucrose	140 mM	9.58 g
D-Mannitol	440 mM	15.3 g
KH_2_PO_4_	20 mM	0.544 g
MgCl_2_	10 mM	0.19 g
1 M HEPES, pH 7.2	4 mM	0.80 mL
0.5 M EGTA	2 mM	0.80 mL
10% BSA	0.4%	8 mL
KOH	n/a	pH to 7.2
ddH_2_O	n/a	*
Total (optional)	n/a	200 mL

*Fully dissolve solid reagents in ~100 mL of ddH_2_O. Use KOH to bring the pH to 7.2 and then bring the solution to a final volume of 200 mL.

Aliquot and store at -20 °C

**11. Working mitochondria assay buffer (1×) with pyruvate and malate**

**Table:** 

Reagent	Final concentration	Quantity or Volume
Mitochondria assay buffer (2× stock)	1×	15 mL
100 mM pyruvate stock solution	10 mM	3 mL
100 mM malate stock solution	5 mM	1.5 mL
ddH_2_O	n/a	10.5 mL
Total (optional)	n/a	30 mL

Stock solutions and buffers should be aliquoted and stored at -20 °. Make this solution fresh on the day of the assay.

**Laboratory supplies**

1. 1.5 mL Eppendorf tubes (Sigma-Aldrich, catalog number: EP022363212)

2. 15 mL centrifuge tubes (Corning, catalog number: CLS430052)

3. 50 mL centrifuge tubes (Corning, catalog number: CLS430828)

4. Pestles (Sigma-Aldrich, catalog number: BAF199230000)

5. Seahorse FluxPak (Agilent)

*Note: This protocol describes experiments performed with Seahorse XF24 FluxPaks. This model has been discontinued and replaced by the Seahorse XFe24 analyzer and FluxPaks (Agilent, catalog number: 102340-100)*

6. 96-well clear flat bottom microplates (Corning, catalog number: 353072)

## Equipment

1. Sorvall Legend X1R centrifuge (Thermo Scientific, catalog number: 75004220)

2. M-20 microplate swinging bucket rotor (Thermo Scientific, catalog number: 75003624)

3. Eppendorf Centrifuge 5425 (Sigma-Aldrich, catalog number: 5405000646)

4. SpectraMax M3 microplate reader (Molecular Devices, catalog number: M3)

5. CO_2_ tank (for *Drosophila* anesthesia)

6. CO_2_ regulator (for *Drosophila* anesthesia) (Flystuff, catalog number: 59-143)

7. CO_2_ fly pad (Flystuff, catalog number: 59-114)

8. CO_2 _blowgun (Flystuff, catalog number: 54-104)

9. Fly brushes (Flystuff, catalog number: 59-204)

10. Fisher Scientific Isotemp incubator, non-CO_2 _(Fisher Scientific, catalog number: 15-103-0514)

11. Seahorse XF24 analyzer (Agilent)

*Note: The current equivalent model Agilent Seahorse is the XFe24. Any Agilent Seahorse will be able to measure oxygen consumption from isolated mitochondria. Volumes and concentrations of reagents should be optimized for the analyzer.*

## Software and datasets

1. WAVE Desktop v2.6 (Agilent, 2018)

2. Prism v10.3.1 (GraphPad, 2024)

3. Microsoft Excel (Office 365, 2024)

## Procedure

**A. *Drosophila melanogaster* preparation**

*Note: Preparation of* Drosophila *may require 1–2 weeks or more to reach the desired age or complete treatment protocols. Plan experiments accordingly.*

1. Collect adult *Drosophila* of the desired age(s) and genotype(s) in fresh food vials.

a. Ensure uniform age of *Drosophila* by removing all adult flies from adults-producing vials the day before collection of experimental flies.

b. Collect experimental flies in new food vials.

c. Maintain consistent numbers in each vial so that fly density does not influence experimental outcomes.

d. Feed flies and incorporate any desired drugs/treatments until flies reach the desired age for isolation.

**B. Preparation of Seahorse calibrant plate and reagents**

*Note: The calibrant plate and sensor cartridge must be prepared the day prior to the mitochondria isolation to allow 16–24 h for the sensor cartridge to rehydrate*.

1. One day before the mitochondria isolation and respiration measurements, prepare the Seahorse calibration plate.

a. Fill each well in the 24-well calibration plate with 1 mL of calibrant solution.

*Note: These instructions are written for a Seahorse XF24 analyzer. The calibration plates, cell culture plates, and sensor cartridges contain 24 wells. Volumes should be adjusted if using a 96-well model.*

b. Place the sensor cartridge in the filled calibration plate. Make sure the sensors are submerged in the calibrant solution.

c. Store the calibration plate with cartridge in a 37 °C incubator (non-CO_2_) for 16–24 h.

*Note: The sensor cartridges arrive sealed and dry. Complete rehydration of the sensors (for O_2_ measurements) is essential for proper assay function.*

2. Prepare all buffers and injection compound stock solutions the day before the mitochondria isolation and respiration measurements.

**C. Mitochondria isolation from whole *Drosophila***

*Note: This section will take approximately 45–60 min to isolate a mitochondria pellet from whole* Drosophila. *Begin thawing all reagents and stock solutions on ice at this time.*

1. Anesthetize flies using CO_2_.

2. Use a brush to move flies (50–100 per strain/condition) into 1.5 mL Eppendorf tubes.

3. Place tubes on ice to keep flies chilled and unconscious while collecting all flies.

*Note: It is not necessary to wash excess cuticle wax prior to the addition of mitochondria isolation buffer.*

**Figure 1.**
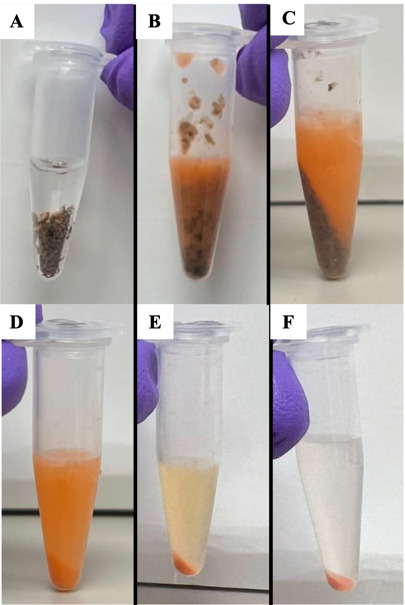
Mitochondria isolation. A–F. Isolation of intact mitochondria by grinding (A) whole flies in (B) isolation buffer and performing (C–F) multiple differential centrifugation steps to clarify a mitochondria pellet.

4. Add 500 μL of ice-cold mitochondria isolation buffer (see Recipes).

5. Homogenize flies using a sterile plastic pestle (Figure 1A, B).

Caution: Perform this step carefully to maintain intact mitochondria. Homogenize using straight up and down motions and gentle pressure. Do not grind the tissue or use a motorized grinder (Figure 1B).

6. Add an additional 500 μL of ice-cold mitochondria isolation buffer.

7. Centrifuge samples at 300× *g* for 5 min at 4 °C. This step will pellet any remaining whole flies, limbs, and cuticles (Figure 1C).

Caution: Carefully check centrifugation speeds for steps C7, 9, 11, and 15. The first two centrifugation steps are slow (300× *g*) to pellet whole cells and debris. The second two centrifugation steps are faster (3000× *g*) to pellet mitochondria.

8. Transfer supernatant to a clean, labeled 1.5 mL Eppendorf tube. The mitochondria will remain in the supernatant. Take care to avoid transferring any debris.

9. Centrifuge samples at 300× *g* for 5 min at 4 °C.

10. Transfer supernatant (Figure 1D) to a clean, labeled 1.5 mL Eppendorf tube.

11. Centrifuge the supernatant at 3000× *g* for 10 min at 4 °C. The mitochondria will be in the pellet after this centrifugation step (Figure 1E).

12. Remove the supernatant.

13. Add 1 mL of mitochondria isolation buffer to the pellet.

14. Resuspend the mitochondrial pellet by gently pipetting up and down with a p1000 pipette.

15. Centrifuge the supernatant at 3,000× *g* for 10 min at 4 °C.

16. Remove the supernatant. Purified mitochondria are in the pellet (Figure 1F).

17. Resuspend the mitochondria pellet in 100 μL of mitochondria isolation buffer.

a. Resuspend the pellet by gently pipetting up and down.

b. Keep the isolated mitochondria on ice.

Caution: Isolated mitochondria should be used for respiration measurements within 4 h of isolation.

**D. Determination of protein concentration in isolated mitochondria**

*Note: This section will require approximately 30–45 min to complete and calculate results. To increase efficiency, begin preparing standards during centrifugation steps in part C.*

1. Create a standard curve using stock BSA diluted in mitochondria isolation buffer. BSA concentrations for the standard curve: 0, 0.2, 0.4, 0.6, 0.8, and 1.0 mg/mL.

2. Dilute isolated mitochondria in isolation buffer. Mitochondria protein concentration must be within the range of the prepared BSA standard curve. Dilutions ranging from 1:10 to 1:100 will typically yield a protein concentration within the linear range of the protein assay.

3. Add 20 μL of Reagent S to 1 mL of Reagent A (both from the Bio-Rad DC Protein Assay kit) to create working Reagent A.

4. Mix the protein assay reagents and diluted standards or mitochondria in a 96-well plate.

a. Set up three separate reaction wells for each standard and mitochondria sample.

b. 5 μL of diluted mitochondria or BSA protein standard.

c. 25 μL of working Reagent A.

d. 200 μL of Reagent B (Bio-Rad DC Protein Assay Kit). Reagent B should be added last. Production of a colored reaction product will begin when Reagent B is added. Work quickly for consistent results.

5. Incubate the protein assay plate at room temperature in the dark for 15 min.

Caution: Color will be stable for up to 1 h after mixing reagents.

6. Measure sample absorbance at 750 nm using a plate reader.

7. Determine protein concentration in mitochondria samples by plotting the BSA standard curve and performing a linear regression.

a. Calculate the mean value for the technical triplicates before performing the linear regression.

b. Make sure to account for dilution of the original mitochondria sample (1:10 or 1:100) when determining the protein concentration of the mitochondria samples.

**E. Respiration measurements with isolated mitochondria**

*Note: Approximately 45 min is required to complete the setup of the Seahorse assay plate, and approximately 60 min is required to complete a Seahorse respiration protocol.*

1. Prepare fresh 1× working mitochondria assay buffer with pyruvate and malate.

*Note: Substrates other than pyruvate and malate may be used in the assay buffer. See General notes.*

2. Dilute mitochondria to 0.1 µg/µL (based on protein concentration assay) in 1× working mitochondria assay buffer (with pyruvate and malate).

3. Obtain a 24-well Seahorse XF24 assay plate.

4. Pipette 50 µL of diluted mitochondria to the bottom of the assay well (5 µg of total protein).

*Note: Use technical triplicates for each unique strain/treatment to ensure reproducibility of results.*

5. Centrifuge assay plate at 3,000× *g* for 30 min at 4 °C in a tabletop centrifuge with a swinging microplate rotor for cell culture plates.

6. Dilute injection compound stock solutions in 1× working mitochondria assay buffer to create working injection compounds (Table 1).

*Note: Perform these dilutions and load the injection ports of the Seahorse assay cartridge (step E7) while the assay plate is in the centrifuge. The assay cartridge should be ready when the centrifugation step is completed.*

a. Perform serial dilutions of injection compound stock solutions to avoid pipetting very small volumes.

b. Injection compound concentrations are calculated based on the desired final concentration in the assay plate. See Table 1 for examples of compound dilutions.

**Table 1.** Injection compounds for Seahorse analyzer

Injection compound	Stock concentration	Working concentration	Injection volume	Final concentration
A. ADP	100 mM	30.66 mM	75 µL	4 mM
B. Oligomycin	10 mM	34.66 µM	75 µL	4 µM
C. FCCP	10 mM	77.32 µM	75 µL	8 µM
D. Rotenone/Antimycin A	5 mM/10 mM	21 µM/42 µM	75 µL	2 µM/4 µM

*Note: See Troubleshooting for suggestions regarding injection compound optimization.*

**Figure 2.**
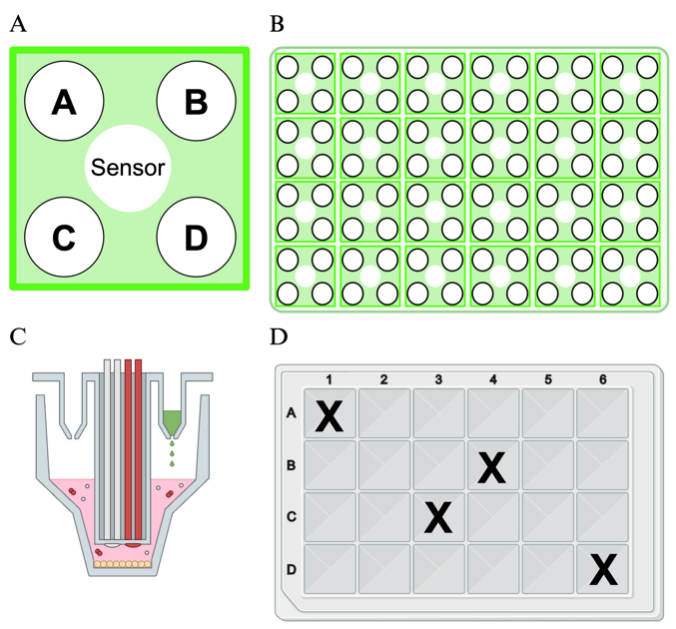
Layout of Seahorse cartridge injection ports and assay plate. A. Arrangement of the injection ports surrounding a sensor in the Seahorse cartridge for an XF24 analyzer. Each well of the 24-well plate (shown in B) contains a group of four injection ports surrounding the sensor. Each section of the injection port will receive 75 µL of working injection compound. C. Side view of an individual injection port and sensor. D. Plate layout for the 24-well plate model. The wells marked with an *X* should be filled with assay buffer only, and no mitochondria, to serve as background controls. The background wells are in the default recommended arrangement used in the WAVE software. They may be changed manually, but users should select multiple background wells in different areas of the plate. Created in https://BioRender.com.

**Figure 3.**
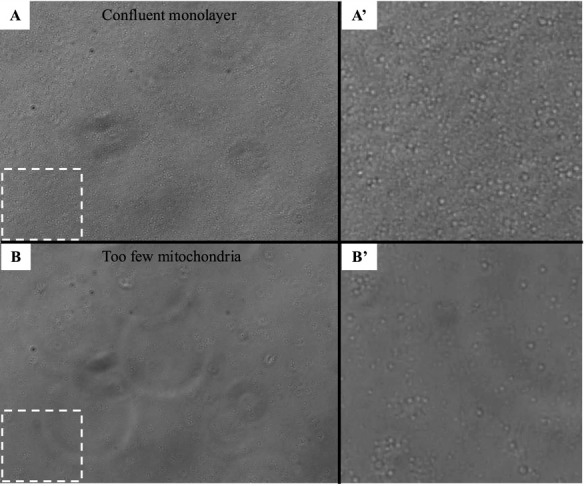
Light microscope image of plated mitochondria after centrifugation of Seahorse assay plate. Panel A and A’ show a confluent monolayer of mitochondria. Panel B and B’ show a plate with a low density of plated mitochondria. A’ and B’ are enlarged images taken from the dashed line boxes in panels A and B.

7. Load 75 µL of each injection compound into the injection ports in the assay cartridge (Figure 2A and B).

8. Use a light microscope to check for mitochondria attachment to the bottom of the Seahorse assay plate. See Figure 3 for an example of a light microscope view of a monolayer of attached mitochondria.

9. Add 450 µL of 1× working mitochondria assay buffer with pyruvate and malate to each assay well. Pipette carefully to the side of the wells. Do not disturb the mitochondria monolayer on the bottom of the plate.

10. Set up the Seahorse XF24 analyzer respiration experiment.

a. Use the WAVE desktop software to program the experiment.

b. Set the desired assay temperature.

*Note: Seahorse analyzer temperatures are dependent on the ambient room temperature of the instrument. The desired experiment temperature should be ~12–20 °C above ambient room temperature. If the desired experiment must be carried out at cooler temperatures, the analyzer may be set up in a cold room.*

c. Identify and label sample groups (technical replicates) before starting the instrument protocol.

*Note: This step is critical for streamlined data analysis.*

d. See Table 2 for an example of the programmed steps for a respiration experiment.

*Note: Each step of the respiration measurement should be repeated 2–3 times to demonstrate stable oxygen consumption rates by the isolated mitochondria.*

**Table 2.** Example Seahorse instrument run protocol

Command	Time	Compound
Calibrate	n/a	n/a
Equilibrate	n/a	n/a
Loop	2×	n/a
Mix	1 min	n/a
Measure	4 min	n/a
Mix	30 s	n/a
Inject A	n/a	ADP
Loop	2×	n/a
Mix	1 min	n/a
Measure	2 min	n/a
Mix	30 s	n/a
Inject B	n/a	Oligomycin
Loop	2×	n/a
Mix	1 min	n/a
Measure	2 min	n/a
Mix	30 s	n/a
Inject C	n/a	FCCP
Loop	2×	n/a
Mix	1 min	n/a
Measure	2 min	n/a
Mix	30 s	n/a
Inject D	n/a	Rotenone/Antimycin A
Loop	2×	n/a
Mix	1 min	n/a
Measure	2 min	n/a
Mix	30 s	n/a
End		

a. First, insert the cartridge and utility plate.

b. Initiate the calibration protocol.

c. After calibration, the Seahorse will eject the utility plate. Remove the utility plate and insert the mitochondria cell culture plate.

*Note: Take care to insert the plate in the correct orientation.*

d. Once the respiration measurements are complete, discard the assay cartridge and the cell culture plate.

## Data analysis

Analysis of the mitochondria protein assay should be performed as quickly as possible while the isolated mitochondria are kept on ice. Linear regression analysis of a standard curve can be performed quickly using Microsoft Excel. Average the absorbance at 750 nm for each standard and sample technical triplicate. Plot the A_750_ vs. the known protein concentration for the BSA standards in an XY scatter plot in Excel (X = protein concentration, Y = A_750_). Include a linear regression on the chart and show the formula. Use the calculated formula to determine the concentration of the diluted mitochondria samples. Be sure to account for any dilution of the mitochondria when determining the concentration in the resuspended mitochondria pellet.

**Figure 4.**
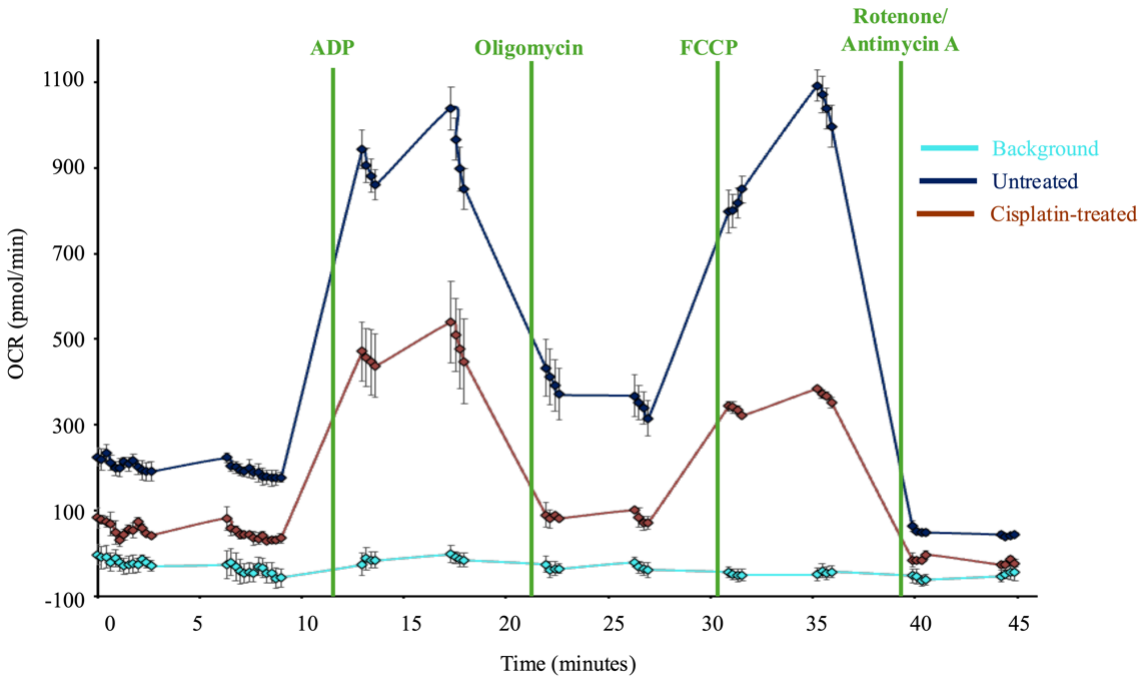
Sample Seahorse data. Graph of oxygen consumption rates (OCR) of isolated mitochondria plated in a 24-well assay and analyzed using a Seahorse analyzer. Injection compounds are noted by vertical lines. Each point on the graph represents an OCR measurement by the instrument (expressed as pmol oxygen consumption/minute). Each point is an average of three technical replicates for each group, with the exception of the light blue line, which represents the four background wells. The error bars represent the standard error of the mean for the technical replicates. The dark blue line is untreated healthy mitochondria, while the dark red line is mitochondria isolated from cisplatin-treated *Drosophila*. Statistical analyses were performed using GraphPad Prism. Treatment groups were compared using Welch’s t-test to compare one group directly to another group at each measurement.

Initial analysis of mitochondria respiration is performed automatically by the Seahorse analyzer WAVE Desktop software. Following completion of the instrument run, WAVE will generate data files containing raw data for oxygen consumption rate (OCR), oxygen pressure (O_2_ mm Hg), and extracellular acidification rate (ECAR), though ECAR is not a useful measurement when assessing isolated mitochondria. The raw data will include a data point for each assay well at every time point measured. Keep these data files for reference. The WAVE software will also automatically average the OCR data for all wells that were grouped as technical replicates. WAVE will also display the average OCR for a group or individual sample well for each measurement cycle. These data may be used to generate data plots in WAVE software. An example of this application is shown in Figure 4. Data from the WAVE Desktop software may be exported to Excel or GraphPad Prism for further analysis.

The most important analysis of Seahorse data can be performed in Microsoft Excel or GraphPad Prism. Mitochondrial function analysis often uses discreet data points to describe the activity of mitochondria in the presence of inhibitors [9]. State 2 respiration is measured prior to the addition of ADP. This value should be low, as the mitochondria do not have a substrate to generate ATP. The addition of ADP creates state 3 respiration, where mitochondria are actively generating ATP. The addition of oligomycin to block ATP synthase creates state 4o. Mitochondria enter state 3u upon the addition of the uncoupler FCCP. OCR following the final injection of rotenone and antimycin A represents non-mitochondrial respiration (state 4). These respiratory states can then be used to calculate other descriptive measurements of mitochondria respiration (See Table 3).

**Table 3.** Mitochondria respiration measurement calculations

Measurement	Calculation	Explanation
Basal respiration	State 3–State 4	Normal function of the mitochondria with substrates available.
Maximal respiration	State 3u	Uncoupled respiration. Maximum OCR for the isolated mitochondria.
Reserve capacity	State 3u–State 3	Measure of the additional potential of mitochondria to increase their oxygen consumption.
ATP-linked respiration	State 3–State 4o	Mitochondria respiration that is generating ATP.
Proton leak	State 4o–State 4	Oxygen consumption that does not generate ATP.
Coupling efficiency	ATP-linked/basal respiration	Estimate of how much of the basal OCR is used to generate ATP.
Respiratory control ratio	State 3/State 4o	Measure of coupling of OCR to ATP synthesis.
State apparent	4 – (ATP-linked respiration/maximum respiratory capacity)	Measure of how close to maximum capacity the mitochondria are.

Calculations of the relevant mitochondria respiration states can be performed in Excel or Prism. Statistical analyses should be performed using software like Prism. The specific tests performed will depend on which samples will be compared. Multiple comparison two-way ANOVA with Holm-Sidak multiple comparisons correction should be used when determining statistical significance between multiple different groups. Unpaired Welch’s t-test may be employed for comparing one strain or treatment to only one other strain. It is useful to perform statistical analyses after calculating the above mitochondria respiration states. Each statistical test will then show if mitochondria from different groups exhibit different respiration at different parts of the experimental protocol. Perform a statistical analysis for each respiration measurement to determine all possible significantly different respiration patterns among the sample groups.

## Validation of protocol

**Figure 6.**
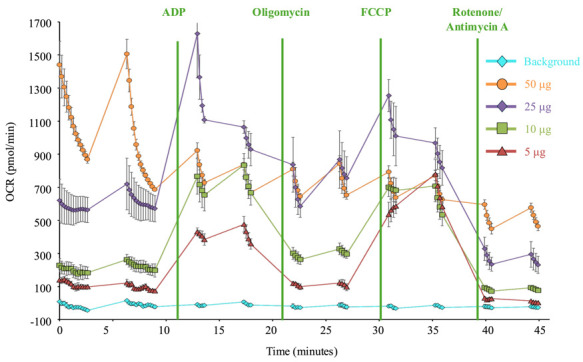
Optimization of mitochondria number. Representative Seahorse respiration data for mitochondria isolated from untreated *Drosophila*. Each line represents a different amount of total mitochondria (determined by protein concentration) plated in the Seahorse assay plate. 50 µg and 25 µg of total protein result in respiration curves that are unstable and rapidly decline during each step, indicating oxygen is consumed too quickly. 10 µg and 5 µg have more stable oxygen consumption rates, but 5 µg shows a more robust response to injection compounds and a smaller standard error of the mean.

This protocol or parts of it has been used and validated in the following research article(s):

- Groen, et al. [16]. Genetic Reduction of Mitochondria Complex I Subunits is Protective Against Cisplatin-Induced Neurotoxicity in Drosophila. Neurobiology of Disease (Figure 6, panel C-K).]
  The protocol described here was validated during the completion of experiments for the above publication. In the 2022 article, we used this protocol to demonstrate that different *Drosophila* strains exhibited different oxygen consumption rates in response to three days of cisplatin treatment, and that improved mitochondria function was correlated with resistance to cisplatin neurotoxicity (see Groen et al. [16], Figure 6C). We validated the isolation of the mitochondria by examining plated mitochondria with a simple light microscope and 20× objective lens to confirm the protocol produced a monolayer in the assay plate (Figure 3). We also produced and fixed a mitochondria pellet, which was prepared and sliced for analysis by transmission electron microscopy. See Figure 5 for representative TEM images confirming that our isolated mitochondria pellets are indeed a highly purified population.
  Validation work for the publication also included testing multiple mitochondria concentrations to identify an optimal quantity of mitochondria that demonstrated a robust response to the injection compounds without completely depleting the oxygen in the sample wells. Once the above validation measures confirmed the procedure yielded an intact, purified population of respiring mitochondria, we completed our analysis of *Drosophila* strain differences with and without cisplatin treatment. At a minimum, we typically perform technical triplicates within an assay plate and complete each set of experiments a minimum of three times. Example data from one Seahorse XF24 analyzer plate is included in Figure 4. The sample data shows two different treatment conditions (with cisplatin and without cisplatin) with three technical triplicates for each treatment group. The data shows respiring mitochondria with a robust response to each of the four injection compounds and low standard error, demonstrating reproducible results across multiple sample wells. The published figure noted above (Groen et al. [16], Figure 6C) uses results from four separate Seahorse assay plates, each with five replicates for each condition. These data demonstrate the protocol described herein yields reproducible data through multiple replicates performed on different days with independent Seahorse assay plates.
  **Figure 5.**
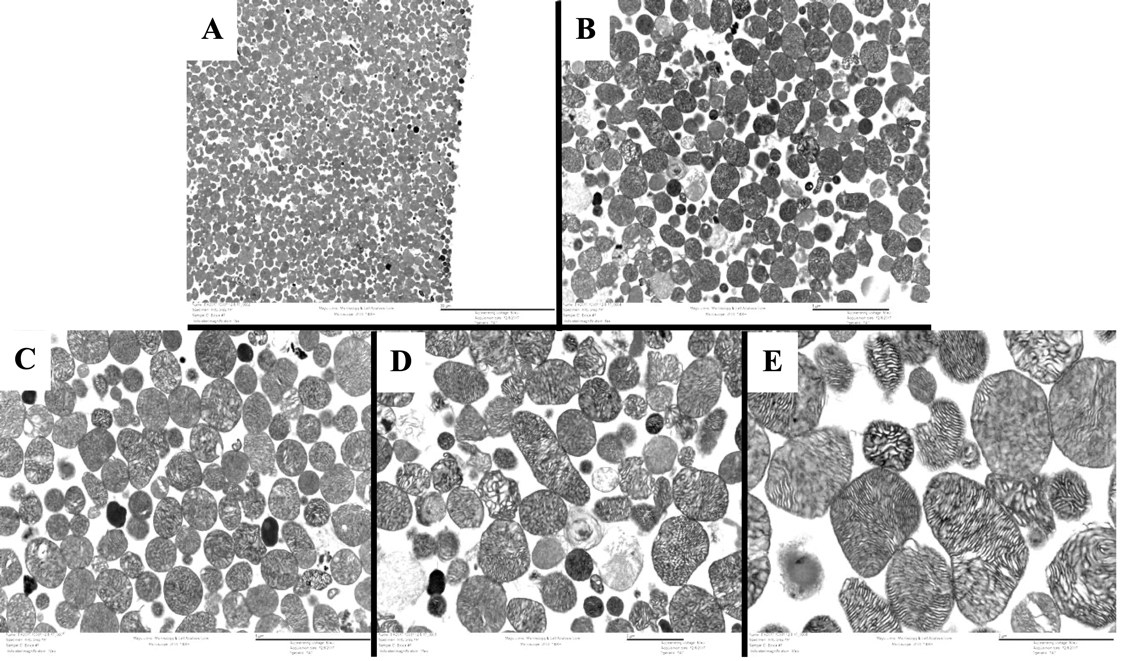
TEM of isolated mitochondria. An isolated mitochondria pellet was fixed and stained for TEM analysis. The pellet was cut after fixation for imaging. Panels A, B, C, D, and E show the isolated mitochondria at 3,000×, 8,000×, 12,000×, 15,000×, and 30,000× magnification, respectively. All images were brightened at 20% to aid visualization.

## General notes and troubleshooting

**General notes**

1. This protocol contains detailed step-by-step instructions for one possible method of respiration measurements. The substrates used in the assay buffer (pyruvate and malate) and the injection compounds (oligomycin, antimycin A, rotenone, FCCP, ADP) may be modified or changed depending on the goals of the experiment. We have described a protocol to analyze electron flow in the mitochondria electron transport chain using substrates for Complex I. This analysis used a pyruvate/malate substrate combination. If researchers desire to assess electron flow from Complex II, succinate and rotenone may be used as an initial substrate combination.

2. An Agilent Seahorse XF24 analyzer was used for the experiments described in this protocol. This analyzer model has since been discontinued and replaced by the Seahorse XFe24 model. Assay-specific details, such as the amount of mitochondria, assay buffer volumes, injection compound volumes, and software setup may have to be modified for the instrument that will be used.

3. This protocol provides step-by-step instructions for a Bio-Rad DC protein assay. Users may use any previously validated protein concentration assay for this section.

4. Seahorse injection compounds are selected based on the desired analysis of the electron transport chain in isolated mitochondria. This protocol shows an example of substrates and inhibitors selected to analyze electron flow through the electron transport chain starting with input to complex I. Pyruvate and malate provide substrates for the conversion of acetyl coA and the citric acid cycle to generate electron carriers for the ETC. ADP provides a substrate for ATP synthesis. Oligomycin A stops the ETC and ATP synthesis by blocking ATP synthase activity. FCCP uncouples the ETC from ATP synthesis and allows assessment of maximal respiratory capacity. Rotenone and antimycin A block the activity of complex I and complex III, respectively.

5. Keep all flies, lysates, and mitochondria pellets on ice or in a 4 °C centrifuge throughout the isolation protocol. Only take samples out of ice to complete pipetting/supernatant transfer steps.

**Troubleshooting**

Problem 1: Mitochondria do not exhibit a change in oxygen consumption rate in response to injection compounds.

Possible cause: Incorrect amount of mitochondria plated, injection compounds are the wrong concentration.

Solution: Try plating multiple amounts of mitochondria and check for a confluent monolayer using a light microscope with a 20× objective. Basal oxygen consumption (before the addition of ADP) should be below 200 pmol/min, as the mitochondria will only be able to use residual ADP in the mitochondria until it is added as an injection compound. Each injection compound concentration will also need to be tested to optimize the OCR response. The suggested range of concentrations to titrate is as follows: ADP (0.5–8 mM), oligomycin (0.5–8 µM), FCCP (2–10 µM), rotenone (0.5–4 µM), and antimycin A (0.5–8 µM).

Problem 2: Unstable OCR measurements. OCR may decline over the 2 min reading for each step of the Seahorse protocol.

Solution: The number of plated mitochondria is likely too high. The oxygen in the assay wells will be used up and cannot replenish fast enough in between mixing steps. Reduce the mitochondria concentration to ensure that the oxygen supply is not exhausted during measurement steps (Figure 6).

Problem 3: High well-to-well variation between technical replicates.

Solution: The mitochondria were likely not mixed well prior to plating. Thoroughly mix the mitochondria in assay buffer by carefully pipetting up and down to completely resuspend and mix the mitochondria.

Problem 4: Mitochondria are not isolated intact.

Solution: Use care when pipetting mitochondria in isolation buffer or assay buffer. Mitochondria should be pipetted slowly with a p1000 pipette to avoid damaging the mitochondria during resuspension steps.

Problem 5: Poor mitochondria function (low OCR readings).

Solution: Perform every step of the isolation on ice or in a 4 °C centrifuge. Work quickly to ensure the Seahorse assay is started before the mitochondria population degrades (mitochondria will remain active for 2–4 h after isolation).
